# Acute effects of a motor coordination intervention on executive functions in kindergartners: a proof-of-concept randomized controlled trial

**DOI:** 10.1186/s40814-022-01125-w

**Published:** 2022-08-17

**Authors:** Petra Haas, Gorden Sudeck, Augustin Kelava, Marcel Cattarius, Marie Meibohm, Johanna Schmid, Eirini Kistoglidou, Caterina Gawrilow

**Affiliations:** 1 LEAD Graduate School & Research Network, Faculty of Economics and Social Sciences, Eberhard Karls Universität Tübingen, Tübingen, Germany; 2 Research Group School Psychology, Department of Psychology, Faculty of Science, Eberhard Karls Universität Tübingen, Schleichstraße 4, 72076 Tübingen, Germany; 3 Education & Health Research, Institute of Sports Science, Faculty of Economics and Social Sciences, Eberhard Karls Universität Tübingen, Tübingen, Germany; 4 Methods Center, Faculty of Economics and Social Sciences, Eberhard Karls Universität Tübingen, Tübingen, Germany; 5 Department of Child and Adolescent Psychiatry, Psychosomatics and Psychotherapy, University Hospital of Psychiatry and Psychotherapy, Tübingen, Germany

**Keywords:** Proof-of-concept, Feasibility of implementation, Acceptance, Executive functions, Motor coordination intervention, Acute physical activity, Kindergartners

## Abstract

**Background:**

Executive functions are pivotal for future academic and social functioning. Causal effects of physical activity on executive functions have been shown in adults. The primary objective of this study was to test the proof-of-concept (i.e., feasibility of implementation and acceptance) of a motor coordination intervention and a sedentary control condition in kindergartners and its preliminary effectiveness on subsequent executive function performance.

**Methods:**

The study used a two-group post-test only design. All children aged between 4 and 7 years old were eligible. One hundred and three children (46% girls; age: *M* = 5.71 years, 95% CI = 5.50 to 5.92) recruited in a middle-sized town in Germany were randomly assigned to a 20-min motor coordination intervention (*n* = 51) or a sedentary control condition (*n* = 52), both of which were conducted in a one-on-one experimenter-child setting in the university or kindergarten. A second blinded-to-condition experimenter assessed the executive function outcomes directly following the conditions. Proof-of-concept criteria were the implementation of the intervention with a moderate-to-vigorous physical activity level assessed via heart rate sensors, and with motor coordination demands, analyzed via video recordings; children’s acceptance via self-reported enjoyment of the conditions; and the post-assessments of executive functions with a behavioral and computerized task.

**Results:**

The motor coordination intervention and the control condition were feasible in a one-on-one setting with kindergartners. The intervention revealed heart rate increases and challenging motor coordination tasks. Children in both conditions indicated they enjoy them. Performance in the two executive function tasks did not differ between children in the motor coordination intervention and the control condition.

**Conclusions:**

A one-on-one experimenter-child setting was feasible to deliver in kindergartners. Future intervention studies should consider pre-testing of executive functions and take into account children’s characteristics as potential moderators, such as motor coordination skills.

**Supplementary Information:**

The online version contains supplementary material available at 10.1186/s40814-022-01125-w.

## Key messages regarding proof-of-concept

- What uncertainties existed regarding proof-of-concept?

It was an open question whether a motor coordination intervention and a control condition are feasible to implement for kindergarten children aged 4 to 7 years.

- What are the key findings for proof-of-concept?

It was feasible to implement an intervention with a moderate-to-vigorous physical activity level and challenging motor coordination tasks and executive function assessments in a one-on-one setting in kindergartners. Both conditions were enjoyable for the children.

- What are the implications of the proof-of-concept findings for the design of the main study?

Pre-tests of executive functions are indispensable to rule out alternative explanations for differences in executive functions between conditions. Motor coordination experiences may have influenced the intervention effects and should be tested in future studies.

## Background

Children engaging regularly in physical activity show improved physical and mental health [1]. Besides these benefits, physical activity is associated with improved cognitive functioning [2]. Executive functions (EFs) are the cognitive processes that seem to benefit most from physical activity, shown in adults [3]. EFs represent the cognitive aspect of self-regulation enabling individuals to exert goal-directed actions [4]. EFs can be separated into three core components: (a) inhibition of prepotent responses, (b) working memory, and (c) shifting [5, 6]. Evidence highlights the predictive power of early EFs in kindergartners (i.e., 4–7 years) for their successful transition to primary school [7] and academic achievement [8]. Thus, this age period may represent a window of opportunity for targeted interventions.

To date, reviews and meta-analyses of randomized controlled trials in children conclude that acute physical activity interventions, implemented as single bout exercise sessions, have beneficial effects on EFs in children [9, 10]. However, further studies in narrowly defined age groups, such as kindergartners, are indispensable [11, 12]. Some studies in children indicate that acute moderate-to-vigorous physical activity interventions enhance EFs, especially inhibition, more than sedentary control conditions [13–15]. In sum, acute physical activity interventions seem particularly promising for improving kindergarten children’s EFs, as they are low cost and easily accessible, yield additional positive effects on children’s physical and mental health [1], and may, thus, be implemented in the daily routines of kindergartens [16].

Various processes have been discussed to explain the mechanisms underlying the benefits of acute physical activity on EF. These mechanisms can be divided into (a) physiological processes and (b) learning processes [2]. Acute physical activity has been shown to induce physiological arousal (e.g., increased P300 amplitude [10],) that may in turn facilitate the allocation of brain resources necessary for EF performance. Moderate-to-vigorous physical activity was most effective for increasing physiological arousal in adults [17].

Second, embodied cognition theories propose that physical activity induces cognitive improvements in children via the bodily interactions and movements in space that accompany physical activity and lead to learning processes [18]. In this way, neurophysiological measures provide evidence for a close interrelation between the cerebellum, activated during complex motor tasks, and the prefrontal cortex, activated during EF tasks [19]. Interventions targeting learning mechanisms refer to qualitative demands of physical activity, such as *cognitive engagement* during physical activity conceptualized as team games or *motor coordination demands* [11, 20]. One study showed improved memory performance after children participated in aerobic team games over and above the effects of mere aerobic exercise [21]. In the same way, an acute motor coordination intervention has been shown to improve subsequent attention more than aerobic exercise in elite sports students [22]. Studies targeting children’s EFs deliver mixed results. Two studies showed improved EFs after acute cognitively engaging physical activity in comparison with sedentary control conditions (in 5–6-year-old kindergartners [23]; in 6–8-year-old school children [24]), while three studies did not find improved EFs after cognitively engaging physical activity compared to sedentary controls (in 5–6-year-old kindergartners [25, 26]; in 10–12-year-old school children [27]) and one study did not reveal advantages of acute cognitively engaging physical activity compared to aerobic exercise, but showed that both physical activity interventions were beneficial for inhibition compared to sedentary conditions [28].

Importantly, positive effects have often been found in group settings [21, 22, 24] but could thus also stem from the social interactions and not be primarily due to the cognitively engaging physical activity. Therefore, standardized implementations in the laboratory are needed to investigate the effectiveness of motor coordination interventions for kindergartner’s EFs. Overall, the studies reported so far indicate that intervention effects of physical activity have often been found in EF tasks focusing on inhibition. In addition, research on cognitive trainings in general has demonstrated greater benefits in *near-transfer* measures (i.e., characterized by high similarity to skills trained in the intervention) than in *far-transfer* measures (i.e., outcome measures that are not highly similar to skills trained in the intervention; [29]).

Taken together, previous research highlights the positive effects of acute physical activity on EFs. Acute motor coordination interventions with moderate-to-vigorous intensity seem most promising for EFs as they address the physiological and learning pathways. To date, randomized controlled studies investigating the effects of acute motor coordination interventions on EFs in kindergartners in one-on-one settings are very limited. The current proof-of-concept trial investigates whether it is feasible to conduct a motor coordination intervention in a one-on-one setting in kindergartners with the specific criteria of incorporating a moderate-to-vigorous physical activity level and motor coordination demands as well as implementing enjoyable conditions. Specifically, the current study has the following *primary objectives*: (1) successful implementation of intervention and control conditions (based on heart rate and video analyses) and (2) acceptance of intervention and control conditions among kindergartners (based on affect and enjoyment). Moreover, the study aims at providing insights into *secondary objectives*, such as (3) preliminary evaluation of effectiveness (i.e., the acute effects of this motor coordination intervention on EFs compared to a sedentary control intervention in kindergartners). We expect that children show higher EF performance immediately after participating in the motor coordination intervention as compared to the control intervention. In addition, this study’s further *secondary objective* (4) was to explore whether there are differential intervention effects depending on the outcome measure (near-transfer vs. far-transfer measure of EF) and children’s previous motor coordination experiences (see Additional file 5).

## Methods

### Sample and design

The proof-of-concept trial used a two-group post-test only design. Children were randomly assigned to either an intervention or a control condition following stratified randomization with four strata (girls/boys × young [4.0–5.9 years]/old [6.0–7.9 years]), starting with a block length of six followed by blocks of four, to account for influences of age and sex. Randomization lists were created using a free online true random number service [30]. These lists were kept confidential and allocation to the intervention took place right before the testing session by experimenter 1 that delivered the intervention (see the “Procedure and experimental manipulation” section). Since the present study aims at testing the feasibility of the conditions, the sample size should be related to the future randomized controlled trial. According to Whitehead and colleagues [31], for an 80% powered main trial with an 0.05 alpha error probability and for detecting a medium effect [2], a sample size of 10 participants in each condition would be appropriate. Since the secondary objective was to assess potential effectiveness, a standard sample size calculation is needed to ensure that there is adequate power ([32], p. 15). An a priori power analysis indicated that 50 participants in each condition would be needed to have 80% power for detecting a medium-sized effect [2] with the 0.05 alpha error probability. The study ended when reaching this sample size.

**Fig. 1:**
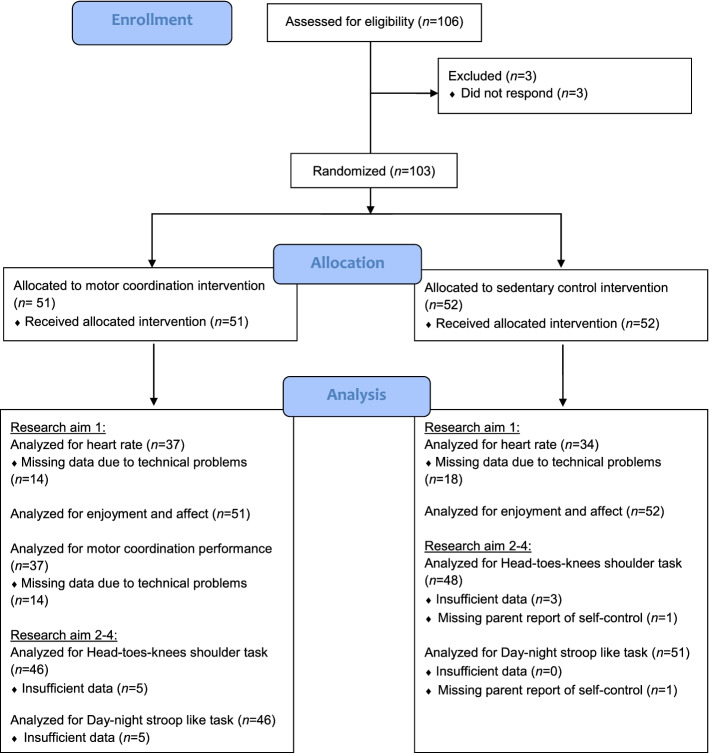
CONSORT flow diagram of the motor coordination intervention and the control condition

Recruitment took place via flyers distributed in preschools, kindergartens, and primary schools as well as sent out via e-mail to students and staff of a university in a middle-sized town in Germany from May to August 2016. All children aged between 4 and 7 years were eligible. The only exclusion criterion was if the parent or legal guardian was unable to provide consent. With their informed consent, the legal guardian also allowed its child to perform physical activity. The CONSORT checklist is provided as Additional file 1, and the participant flow diagram is presented in Fig. 1 [32]. The sample was characterized by a high parental education level; 81% of children had two parents with a university entrance qualification. The majority (i.e., 92%) of families spoke German at home.

Parents gave written informed consent before study participation, and children provided verbal consent before study participation. Children received a 7€ voucher for a local bookstore. The study was approved by a local ethics committee (Az 2016/0218/01) with written informed consent from all legal guardians of the subjects in accordance with the Declaration of Helsinki and verbal consent to participate by the children.

### Outcome measures

#### Heart rate and motor coordination demands

To evaluate the implementation of the conditions, we measured children’s *heart rate* during the intervention and control conditions. Video recordings of children in the motor coordination intervention served to indicate motor coordination demands.

Children’s heart rate (HR) was assessed using an electrocardiograph device (ekgMove; Movisens®, Karlsruhe, Germany) as an objective indicator of physical activity intensity. The device (62.3 mm × 38.6 mm × 10.5 mm) was placed at the children’s thorax, to the right of the sternum, with adhesive disposable electrodes and a band aid. The raw data were aggregated into 15-s epochs to include even bouts of short physical activity [33], and the average HR per epoch was extracted using DataAnalyzer [34]. The raw data was then screened for artifacts, defined as either (a) a HR below 60 beats per minute (bpm; e.g., [35]) or above 250 bpm [36] or (b) multiple consecutive records with the very same HR. In both cases, single records were deleted. If multiple records (i.e., > 30%) for one child were missing, the whole recording was excluded from the analyses. Consequently, valid data consisted of mean HRs ranging from 60 to 250 bpm for every 15-s epoch. We calculated mean HRs per child during the session based on the validated HR data. Since we aimed at inducing a moderate-to-vigorous physical intensity with the intervention, we analyzed children’s mean HR during the session in relation to children’s maximal HR (i.e., relative HR max). Children’s maximal HR was based on age-dependent norm values and set to 193 bpm [37]. Moderate physical activity refers to 55–70% of maximal HR, while 70–90% of maximal HR relates to vigorous physical activity in children [38]. Thus, benchmarking scores of children’s HR in the motor coordination intervention were set to mean HR equal or greater than 106 bpm. For children in the control condition, we set benchmarking scores at mean HR below 106 bpm.

To establish the proof-of-concept for implementing challenging motor coordination demands in the motor coordination intervention, children were videotaped during the intervention. One trained rater evaluated how often children performed a round correctly and passed the difficulty levels of each motor coordination game. One round was rated as correct if all criteria were fulfilled, and else as incorrect (see criteria in detail in Additional file 2). After completing three correct rounds, difficulty level 2 was introduced. Inter-rater reliability was calculated by having a second rater with partial overlap among rating subjects (ICC(A,2) = 0.99; *p* < 0.05; *n* = 6 children; [39]). The benchmarking score for challenging motor coordination demands of the intervention was defined as the majority of children in the intervention condition reaching difficulty level 2.

#### Affect and enjoyment

To evaluate the acceptance of the intervention and control conditions, we assessed children’s current *affect* and *enjoyment*. *Affect* was assessed with two items representing valence and arousal on a 5-point scale via the Self-assessment Manikin Scale ([40]; see Additional file 3) before and after the condition took place. Benchmarking scores for evaluating the acceptance of the conditions were defined at a score of lower than 3 for valence (representing positive affect) and at a score of 2 or higher for arousal (indicating middle-to-low arousal). Children’s *enjoyment* of the conditions was assessed with four items: enjoyment/fun, difficulty, exhaustion, and motivation to repeat [41], answered on a 3-point ordinal scale with child-oriented graphics [42]. Acceptability of the conditions was inferred when enjoyment and motivation items were evaluated at a score equal or higher than 2 (i.e., indicating middle-to-high enjoyment and motivation to repeat the intervention on the 3-point scale) and when difficulty and exhaustion items were evaluated a score equal or lower than 2 (i.e., indicating middle-to-low difficulty and exhaustion on the 3-point scale).

#### Executive functions

Two executive function tasks were carried out in order to capture (a) behavioral expressions of EFs that also represent a near transfer outcome since the required responses were close to the intervention’s content, using the head-toes-knees-shoulders task, as well as (b) verbal expressions of EFs that represent a far-transfer outcome since the required responses were not very close to the intervention’s content, using the day-night Stroop-like task. Between these two tasks, a small-to-moderately high significant correlation (*r* = 0.29; *p* < 0.05) has been reported by previous research in a sample of kindergartners (*M* = 5.66 years; *SD* = 0.32 [43]), hence suggesting convergent validity.

The *head-toes-knees-shoulders task* (HTKS) is a standardized behavioral test that measures “self-regulation as the manifestation of EF skills,” such as working memory, and inhibition, manifested in gross motor actions ([44], p.605). Children are instructed to perform the opposite movement of what the experimenters tell them to do (e.g., touch their head when asked to touch their feet). The task includes three block items of increasing difficulty, each with 10 test items. Children have to achieve at least 4 points to continue on to the next block. Experimenters rate task performance in situ and children are videotaped during the task that blind raters can code the responses as correct (2), incorrect (0), or corrected response (1). Response accuracy (i.e., sum score, range 0–60) serves as the indicator of EF performance. Mean ratings of the blind raters are used in the analyses, with the exception of 5 children for whom in situ experimenter ratings of blinded-to-condition experimenters were used due to technical problems with videotaping. Inter-rater reliability was calculated in this study by having three blind raters code participants with partial overlap among rating subjects (Krippendorff’s *α* = 0.96; *n* = 50; [45]). The high inter-rater reliability is in line with previous findings (Cohen’s *κ* = 0.90; [44]). Prior studies have shown that the HTKS has good test–retest reliability (*r* = 0.60–0.74 at a time-lag of 5.7 months) as well as high construct validity with other EF assessments [43].

To assess EFs via a far transfer measure, we used the computer-based, child-adapted *day-night Stroop-like task* (DNS) [46, 47]. Children are instructed to name the opposite of what they actually see (e.g., say “girl” for a picture of a “boy”) after having learned about four picture pairs. The stimuli are then presented on a laptop using the software DirectRT [48]. Experimenters instruct the children to answer as quickly and correctly as possible. Responses are categorized as correct (2), incorrect (0), or corrected errors (1). Response accuracy serves as the indicator of inhibition capacity. Sum scores across both blocks (range 0–96) were used in the analyses. Prior studies have shown that the DNS is correlated with other inhibition measures [49] and demonstrated good reliability (*r* = 0.84; *p* < 0.05; time-lag of 2 weeks, *n* = 22 children; [50]).

#### Motor coordination experience

Children’s previous level of motor coordination experience was assessed using a short parent questionnaire for preschool children ([33]; see Additional file 4). The questionnaire has been developed and validated to assess physical activity behavior in preschool children. For seven items, parents rated how often their child regularly engages in the indicated activity (e.g., *play with a ball*) on a 5-point ordinal scale (1, *never*; 5, *every day*). Parent ratings for each item were classified as an indicator of high (+ 1) or low (− 1) habitual experience with the corresponding activity depending on the indicated frequency. Sum scores for all item responses were calculated for each child (range − 7 to + 7). The questionnaire scores have been shown to be positively associated with preschool children’s objectively recorded physical activity in daily lives [33] as well as with performance in a motor test [51]; these studies reflect the validity of the questionnaire.

#### Background variables

Parents provided demographic data on their child (e.g., sex, age, height, weight) and on themselves (e.g., mother tongue, education level, other parent’s education level). Children’s body mass index (BMI; kg/m^2^) was calculated from the parent’s report. To control for pre-existing differences in children’s self-regulation, we used parents’ ratings of their child’s dispositional capacity for self-control via the brief *Self-control Scale* [52, 53] on a 5-point Likert scale (*1, not at all; 5, exactly*), with higher sum scores representing lower self-control (range 13–65). The questionnaire exhibited adequate reliability (i.e., Guttman’s *λ*_2_ = 0.85), in line with previous results [53]. Experimenters recorded the setting (university vs. kindergarten or primary school) and time of the session (morning: start before or at 12 pm vs. afternoon: start later than 12 pm).

### Procedure and experimental manipulation

The study consisted of one session (52 min) that took place in a one-on-one experimenter-child setting in a room at the university (*n* = 77 children) or kindergarten (*n* = 23 children) or primary school (*n* = 3 children). If the child felt uncomfortable, parents sat in the back of the room (*n* = 10 children). Experimenters were six trained research assistants. The session consisted of two parts, with part 1 including the experimental manipulation (i.e., intervention or control condition) and part 2 including the assessment of the dependent variables (i.e., EF tasks). The experimenter for part 2 was blind to the experimental condition to avoid any prompting in line with the hypothesized outcome. After briefly introducing the study goals, experimenter 1 asked the children about their current *affect* and attached the electrocardiographic device for assessing the *heart rate*. Next, the intervention/control condition started; this portion of the experiment was videotaped in order to monitor the feasibility of implementing challenging motor coordination tasks in the motor coordination intervention.

In the intervention condition (20 min), children performed four different motor coordination games embedded in a child-appropriate story (see Additional file 2). Each game was developed to impose moderate-to-vigorous intensity (55–90% of maximal heart rate; [38]) and to demand a wide range of basic motor skills, such as locomotion (e.g., jumping, running) and object control (e.g., throwing or bouncing a ball [54];). The games were *adaptive*, with children receiving more difficult tasks with increasing performance (i.e., two levels per game). Each game started with a short introduction, then the child had 3 min to perform the instructed activities. For each round within a game, the child received a stamp. The duration of 20 min has been shown to improve subsequent EFs in adults [55].

In the control condition (16 min), the experimenter read a story to the child and asked easy open-ended questions at regular intervals. The child received a stamp for each answer or comment to the questions. After the intervention/control condition, experimenter 1 left and experimenter 2 returned. The child indicated its current *affect* and was asked about its *enjoyment* of the tasks. Then, the *HTKS* was performed followed by the *DNS*.

### Statistical analyses

All analyses were conducted with SPSS Statistics 26®. In cases, where the sample size was higher than 30, an approximative normal distribution was assumed, due to the central limit theorem ([56], p. 297–311). Metric variables following a normal (or approximative normal) distribution were described with mean and standard deviation. Nominal variables were described with frequency outputs. To assess the primary outcomes (1–2), descriptive statistics of the relevant variables were observed and compared to the aforementioned benchmarking scores. Furthermore, primary outcomes were assessed by comparing the two groups, relying on the 95% confidence intervals of differences between groups. For the secondary objectives (3–4), preliminary intervention effects were evaluated based on 95% confidence intervals of adjusted mean differences between the control and intervention groups. Therefore, linear regression models were run to observe adjusted mean differences of the outcome variables (i.e., HTKS, DNS) between the two condition groups, after controlling for children’s age, sex, motor coordination experience, and self-control. Correlation analyses were run to detect convergent validity between both EF measures. Exploratory analyses addressed differences in intervention effects depending on the outcome measure (i.e., near-transfer measure of EF = HTKS vs. far-transfer measure of EF = DNS) by comparing standardized regression coefficients (see Additional file 5). In addition, we included levels of motor coordination experience as a predictor and moderator of the intervention effects (see Additional file 5). All predictors were centered at the grand mean or dummy-coded, and we screened for multicollinearity between predictors.

## Results

### Implementation and acceptance (primary outcomes)

The total sample consisted of *N* = 103 children (46% female; age: *M* = 5.71 years, 95% CI = 5.50 to 5.92; *n* = 84 children attending German kindergarten; *n* = 18 children attending German primary school). From the total sample, *n* = 51 children were randomly assigned to the intervention condition and *n* = 52 children to the control condition (see the participant flow diagram in Fig. 1). Baseline characteristics seemed to be relatively equally distributed in the two groups. Demographics and testing characteristics can be seen in Table 1.

**Table 1:** Descriptive statistics for sample and testing characteristics of the two conditions (control vs. intervention)

Variable	Control		Intervention	
	*n*	*M* (*SD*)	*n*	*M* (*SD*)
*Sample characteristics*				
Age [years]	52	5.66 (1.06)	51	5.76 (1.10)
Sex				
Female	25		22	
Male	27		29	
Body mass index	47^a^	14.76 (1.51)	45^a^	14.73 (1.23)
Motor coordination experience	52	0.33 (3.08)	51	0.67 (3.49)
Self-control	51^a^	43.41 (9.16)	51	43.76 (8.41)
*Testing characteristics*				
Setting				
University	42		35	
Kindergarten/primary school	10		16	
Time of session				
Morning	22		22	
Afternoon	30		29	

^a^Non-reported values are missing

To ensure successful implementation of the motor coordination intervention, HRs during the two conditions were compared for 71 children providing valid data (see Table 2 for results, see the “Methods” section for HR data processing). As expected, mean HRs during the intervention condition were higher than during the control condition, indicating increased physiological arousal in the intervention. According to the 95% CI, heart rate scores ranged in line with the bench marked scores for both groups. The mean HR in the intervention condition (i.e., 127 bpm) corresponded to 65% of the estimated maximal HR [37], with the 95% confidence interval ranging approximately from 64 to 68% of the estimated maximal HR. In video analyses (relying on a subgroup of children in the intervention condition, *n* = 37), the rater identified that on average 80% of the children reached level 2 of the coordination games. In addition, 55% of children reaching level 2 also performed the games as instructed.

**Table 2:** Descriptive and test statistics for proof-of-concept criteria (feasibility of implementation and acceptance; primary outcomes) of the two conditions and of the difference scores between conditions

Variable	Control		Intervention		Diff
	*n*	*M* (*SD*)	*n*	*M* (*SD*)	*M* Diff (95% CI)
Heart rate	34^b^	101.05 (12.18)	37^b^	127.30 (10.61)	26.25 **(20.85; 31.65)**
Enjoyment	52	2.81 (0.49)	51	2.75 (0.52)	− 0.63 (− 0.26; 0.14)
Perceived difficulty	52	1.46 (0.64)	51	1.84 (0.64)	0.38 **(0.13; 0.63)**
Perceived exhaustion	52	1.33 (0.51)	51	2.10 (0.78)	0.77 **(0.51; 1.03)**
Motivation to repeat	52	2.46 (0.78)	51	2.16 (0.83)	− 0.31 (− 0.62; 0.01)
Affect					
Valence pre	52	1.17 (0.38)	51	1.61 (1.13)	0.43 **(0.11; 0.76)**
Valence post	52	1.38 (0.75)	51	1.57 (1.01)	0.18 (− 0.16; 0.53)
Valence post–pre	52	0.21 (0.67)	51	− 0.04 (0.85)	− 0.25 (− 0.55; 0.05)
Arousal pre	51^a^	2.63 (1.23)	51	2.63 (1.43)	0.00 (− 0.52; 0.52)
Arousal post	52	2.71 (1.35)	51	2.59 (1.33)	− 0.12 (− 0.65; 0.40)
Arousal post–pre	51^a^	0.08 (0.69)	51	− 0.04 (1.18)	− 0.12 (− 0.50; 0.26)

*Diff* Difference between conditions, in bold if *p* < 0.05

^a^Non-reported values are missing

^b^invalid values are missing

To further investigate feasibility, children indicated their affect and enjoyment after participating in the intervention or control condition. In sum, children reported high positive affect and were moderately aroused before and after the intervention and control conditions. Children indicated overall high enjoyment of the conditions (*M* = 2.78; 95% CI = 2.68 to 2.88, on a scale from 1 — *not at all* to 3 — *very much*) and did not differ in their enjoyment of both conditions. Concerning the set benchmarks, based on mean scores (see Table 2), the criteria are met. However, children in the intervention condition perceived this condition to be more difficult and more exhausting than children in the control condition. Children in both conditions were inclined to repeat the condition, but children in the intervention condition were to a little degree less willing to repeat the intervention than children in the control condition (see Table 2).

### Intervention effects on executive functions (secondary outcomes)

Performance in EF measures ranged from 65% mean accuracy in the HKTS to 74% mean accuracy in the DNS. Scores of the HTKS and DNS were moderately correlated, *r*(92) = 0.458; *p* < 0.05, indicating convergent validity of the two outcome measures. However, differences are apparent given the only moderate size of the correlation; this underlines their function as near-transfer and far-transfer measures.

Children with invalid test scores due to non-comprehension or refusal to complete the EF task were excluded from the regression analyses testing the intervention effects (secondary outcome; see Fig. 1). In addition, one parent did not indicate his/her child’s self-control and this case was also excluded. The final dataset for the analysis of the HTKS consisted of *n* = 94 children. The excluded children did not differ from the remaining sample with respect to their assignment to the conditions or with respect to background variables. The final dataset for the analysis of the DNS consisted of *n* = 97 children. These children did not differ from the remaining sample with respect to background variables, but all had been assigned to the intervention condition. Since the DNS was assessed at the end of the testing session, this finding may be attributed to these children’s tiredness.

#### Head-toes-knees-shoulders task

Adjusted mean differences and their 95% confidence intervals revealed no main effect of condition on performance in the HTKS (secondary outcome 3; see Table 3). Thus, children who participated in the intervention condition did not show higher EF performance compared to children in the control condition, when controlling for age, sex, motor coordination experience, and self-control. There was no multicollinearity between predictors.

**Table 3:** Descriptive and test statistics for executive function performance (secondary outcomes) of the two conditions and of the adjusted difference between conditions

Variable	Control		Intervention		Adj. Diff
	*n*	*M (SD)*	*n*	*M* (*SD*)	*M* Adj. Diff (95% CI)
Head-toes-knees-shoulders task	48	40.76 (12.07)	46	41.38 (13.19)	0.22 (− 3.44; 3.87)
Day-night Stroop-like task	51	74.06 (18.47)	46	74.37 (18.99)	− 0.62 (− 6.86; 5.82)

Invalid values are missing; *Adj. Diff* Adjusted difference between conditions accounting for children’s age, sex, motor coordination experience, and self-control

#### Day-night Stroop-like task

Intervention and control conditions did not differ in their DNS performance, when controlling for age, sex, motor coordination experience, and self-control (secondary outcome 3; see Table 3). Hence, children in the intervention condition showed no higher performance compared to children in the control condition. There was no multicollinearity between predictors.

## Discussion

This randomized proof-of-concept trial investigated whether it is feasible to conduct a motor coordination intervention and a control condition in a one-on-one setting in kindergarten children aged 4 to 7 years (primary outcomes 1–2). The motor coordination intervention was successfully implemented with moderate-to-vigorous intensity and challenging motor coordination tasks. Both conditions were enjoyable for the children. Moreover, the study aimed at providing insights into the acute effects of a motor coordination intervention on EFs compared to a sedentary control intervention (secondary outcome 3). In contrast to our hypothesis, children who were randomly assigned to and participated in the motor coordination intervention did not differ in their EF performance right after the intervention from children who participated in the control condition.

### Proof of concept: primary outcomes

This study showed that it was feasible to implement a motor coordination intervention lasting 20 min followed by two EF assessments in a one-on-one setting in children aged 4 to 7 years. The intervention was feasible in various settings (university, kindergarten, or primary school) and at various time points (morning, afternoon). Children’s mean HR during the motor coordination intervention indicated a moderate-to-vigorous intensity, which has been previously shown to benefit EFs [14], and was higher than mean HR in the sedentary control condition. To introduce motor coordination demands for children in the motor coordination intervention, we implemented adaptive games meaning that children received more difficult levels of a motor coordination game after succeeding in the first level. Video analyses showed that the majority of children reached level 2 of the coordination games, while only half of the children reaching level 2 also performed the games as instructed. So, the current motor coordination intervention allowed most children to show their mastery in level 1 and at the same time remained challenging in level 2. Both conditions were enjoyable for the children, but children in the motor coordination intervention reported this condition to be more difficult and exhausting than children in the control condition. Importantly, this difference was intended when developing an intervention with moderate-to-vigorous physical activity level and motor coordination demands. Children’s affect ratings pre- and post-intervention documented that children felt well during the testing session in both the motor coordination intervention and control condition. Resulting from these criteria for proof-of-concept (i.e., heart rate, video rating of motor coordination demands, affect, and enjoyment), the motor coordination intervention was successfully implemented among the present study sample and should address the physiological and learning pathway of physical activity benefits for EF. Further on, the assessments of EF were feasible since there were only few missing observations due to incomprehension of the EF tasks. Ceiling or floor effects in the EF tasks were not present, as evidenced by accuracy scores in line with previous findings (HTKS: [43]; DNS [57]). Future full-scale randomized studies could use the present motor coordination intervention and the proposed EF tasks to analyze the effectiveness and mechanisms of exercise on children’s EF while considering the limitations and interpretations of the present preliminary findings (see next sections).

### Intervention effects on executive functions: secondary outcome

The preliminary finding that a motor coordination intervention did not improve the inhibition component of EFs more than a sedentary activity in kindergartners stands in contrast to other studies with kindergartners, children, or adolescents showing cognitive benefits after acute cognitively engaging physical activity [21–24, 28]. However, no positive effects of activity on EF were also shown in certain studies among kindergarten and school-aged children [25–27]. Importantly, the preliminary finding of no EF differences after the conditions cannot be attributed to existing differences in children’s age, sex, body mass index, parent-reported self-control, levels of motor coordination experience, nor to differential effects of the conditions on children’s enjoyment or changes in affect from pre- to post-parent-reported. Moreover, our study design with a one-on-one experimenter-child setting rules out alternative explanations due to social interaction influences in a group setting [11] or bias from an unblinded experimenter assessing children’s EFs [58].

Further explanations for the lack of positive effect of physical activity with motor coordination demands on EFs could be due to (a) differential effects in kindergartners and (b) the lab-based setting of our intervention. First, it could be that the positive activity effects found for adults and older children do not generalize to younger children aged 4 to 7 years. Moreover, kindergartners differ considerably from each other with regard to their current developmental state in terms of cognitive and motor skills [59]. These individual differences in EFs as well as motor coordination skills may moderate the effects of a motor coordination intervention on EFs. To account for effects due to this sample’s age range of 4 years, we controlled for a moderating influence of age that was not significant. We also controlled for children’s self-control as a proxy for EFs, and self-control did not moderate the effects of condition on EF performance. In line with previous findings [60], we also conducted exploratory analyses that included children’s level of motor coordination experience as a moderator for the activity effects on EFs (see Additional file 5 and the next section).

Second, the lab-based setting of our motor coordination intervention may differ from prior studies that found a physical activity effect on EFs [24]. The model on the influence of skill acquisition for cognitive benefits proposes that affective and motivational features of an intervention, such as social interactions, modeling, and cooperative learning, are critical for cognitive benefits [61]. Social interactions during physical activity seem to promote EFs more than engaging in physical activity alone, shown in adults [62]. In the present study, children performed the motor coordination intervention with only the experimenter present who did only once model the behavior but not actively participate her/himself. This setting may have eliminated a necessary factor for the activity effect on EFs, which are social interactions [21, 61]. Future studies should address this issue by examining the mechanisms leading to activity effects on EF comparing single and group settings.

### Exploratory findings of differential effects (preliminary effects of secondary outcomes)

In exploratory analyses, we tested whether our intervention had differential effects depending on the outcome measure and a child’s previous motor coordination experience (see Additional file 5). Children with very low levels of motor coordination experience performed worse in the HTKS after the motor coordination intervention compared to the control condition. Children with very high levels of motor coordination experience performed better in the HTKS after the motor coordination intervention compared to the control condition. Importantly, this was an exploratory finding and the effect was only true for children with very low (i.e., < 2.12 *SD*) or high levels (i.e., > 1.60 *SD*) of motor coordination experience.

This result may be explained by an *optimal challenge point*, which means that characteristics of the physical activity intervention (e.g., motor coordination demands) optimally match characteristics of the child (e.g., motor coordination experience) to result in cognitive improvements [60]. In light of the close interrelation between the cerebellum, activated during motor coordination tasks, and the prefrontal cortex, activated during EF tasks [19], the cerebellum might be only activated during motor coordination exercises if a certain amount of motor coordination experience is present. Children with very low levels of motor coordination experience might instead show different neurophysiological activation patterns. Support for this hypothesis stems from studies showing that children with developmental coordination disorder exhibit under-activation of cerebellar networks compared to normally developed peers [63]. In addition, one study in school children (*M* age = 11.29 years) showed that individuals’ fitness level moderated whether an acute physical activity intervention benefitted EFs compared to a sedentary control condition [27]. So, it may be that in the present study children did not generally benefit from the intervention since they had low fitness levels or low experience with motor coordination tasks.

Importantly, different levels of motor coordination experience were linked to improved inhibition performance assessed in a behavioral task (i.e., HTKS), but not in a computerized task (i.e., DNS). These tasks require either motor or verbal inhibition [64]. Consequently, the effect of children’s motor coordination experience was limited to a *near-transfer* outcome measure requiring full-body motor responses that was strongly linked to the intervention content. In line with embodiment theories, it could be that the movement-induced activation of cognitive networks primes subsequent motor performance in the EF task [18].

### Limitations

This proof-of-concept trial is not without limitations. First, we did not measure EFs in a pre-test due to missing availability of EF tests that are suited for repeated assessments in short time periods for children aged 4 to 7 years at the time of running the study in 2016 (e.g., prior studies assessed 6-month retest reliability of HTKS [43] or tested battery of EF tests [65]). We had concerns that learning effects would undermine potential intervention effects when assessing EFs repeatedly within short time periods (i.e., 20 min of intervention/control condition for separating pre and post-test assessments) or be biased by children’s fatigue. Therefore, randomization was used to establish equally distributed groups in terms of pre-existing EF abilities. Additionally, important covariates of EFs were controlled to be equally distributed in both conditions. However, motor coordination experiences may possibly be related to EF performance regardless of the intervention or control condition. Future full-scale studies should implement a pre-test of EFs to rule out alternative explanations for differences in post-test EFs between the conditions. By using a pre-post-test design with intervention and control conditions, changes in EFs can be attributed to the respective condition and a training effect. Second, we assumed that a motor coordination intervention would be more beneficial for improving EFs than a physical activity intervention without motor coordination demands, as the former addresses both the physiological as well as learning pathways. However, to further test this assumption, studies are needed that directly test the assumed mechanisms and compare physical activity interventions with varying intensities and varying motor coordination demands. Third, future studies may wish to corroborate our findings by assessing motor coordination objectively (e.g. [66]).

Fourth, the present study implemented an acute intervention, with the goal of developing an easily implementable intervention for kindergarten or school settings. Moreover, testing an acute intervention allowed us to control for possible beneficial effects due to maturation processes observable in chronic interventions. However, chronic interventions may accumulate the effect of physical activity over time and may thus exhibit higher effectiveness for improving EFs. A recent review showed that chronic physical activity interventions, in particular, benefitted EFs in preadolescent children (6–12 years; [67]). One study with kindergartners showed that motor coordination interventions repeated twice a week for 8 weeks were successful in improving inhibition [68]. Further on, our motor coordination intervention targeted a wide range of basic motor skills, such as locomotion (e.g., jumping, running) and object control (e.g., throwing or bouncing a ball [54]). Diverse training may only lead to improved EFs in the long run [69]. Future studies assessing both the short-term and long-term effects of the current motor coordination intervention in one study could shed light on the effectiveness and sustainability of effects.

Fifth, we tried to develop the motor coordination intervention and the control condition as similarly as possible, but the duration of the two conditions varied slightly (intervention took 4 min longer than the control condition) and children in the motor coordination intervention were less inclined to repeat the condition, which may reflect their momentary higher exhaustion level than children in the sedentary control condition. Sixth, an a priori power analysis that also considers testing covariates besides the intervention effects should be included in full-scale studies. In addition, future studies should recruit samples with diverse socio-economic backgrounds as the present study is limited to children from highly educated families.

## Conclusion

To our knowledge, this is one of the first proof-of-concept trials to investigate the feasibility, acceptance, and preliminary acute effectiveness of a motor coordination intervention on EFs among kindergartners aged 4 to 7 years. The strengths of the current study are its implementation of a motor coordination intervention that addresses physiological arousal and motor coordination demands in a one-on-one experimenter-child setting and the assessment of EFs by blinded-to-condition experimenters. Importantly, our motor coordination intervention did not generally benefit kindergartners’ EFs more than a sedentary control condition shown in preliminary analyses. The results were the same for a near-transfer and far-transfer measure of EF. Future full-scale studies should implement pre-tests of executive functions to derive conclusions on changes in EFs that are validly attributable to the experimental manipulation. Besides, future studies should take into account individual characteristics that may moderate intervention effects, such as motor coordination experiences optimally assessed by objective test batteries.

## Supplementary Information

**Additional file 1.** CONSORT checklist extension for pilot and feasibility trials. Pilot trial is described in detail following the CONSORT guidelines.

**Additional file 2.** Description of the motor coordination intervention and criteria for difficulty levels. Intervention is described in detail as well as the specific criteria for Level 1 and Level 2 of each game in the motor coordination intervention.

**Additional file 3.** Affect measure. The assessment of affect is described in detail.

**Additional file 4.** Motor coordination experience. Questionnaire items to assess motor coordination experience are described in detail.

**Additional file 5.** Further analyses on the acute effectiveness of the conditions. Regression analyses of the conditions on executive function performances are described in detail.

## Data Availability

The datasets used and/or analyzed during the current study are available from the corresponding author on reasonable request.

## Abbreviations

EF: Executive function
DNS: Day-night Stroop-like task
HTKS: Head-toes-knees-shoulders task
HR: Heart rate
