# Supplementary material for: Acute effects of a motor coordination intervention on executive functions in kindergartners: a proof-of-concept randomized controlled trial
Source: Pilot Feasibility Stud. 2022 Aug 17;8:185. doi: 10.1186/s40814-022-01125-w (PMC9382724; doi:10.1186/s40814-022-01125-w)
Supplement: Supplementary file 2 — Additional file 2. Description of the motor coordination intervention and criteria for difficulty levels. Intervention is described in detail as well as the specific criteria for Level 1 and Level 2 of each game in the motor coordination intervention. [file 40814_2022_1125_MOESM2_ESM.docx]

# Additional file 2

# Description of the motor coordination intervention

The motor coordination intervention started with a short story about two friends, a rabbit and a hedgehog, who encounter a bear with a broken leg and want to help him by collecting food for him. Each game was embedded in this story such that the participating child always had to fulfill a task in order to collect some food for the bear. In addition, the child was instructed to perform all of the following games as fast as a rabbit. The child received a stamp for each round within each game, and the goal was to collect stamps across all four games in order to receive a gift at the end of the session. After this short introduction, four different games (5 min each) with an increasing difficulty level were completed. The experimenter ensured that each child performed at least 3 minutes of physical activity in each game by using a stop watch to exclude instruction time. The games were performed in a 2.5m x 5 m area. The following paragraphs explain each game and its difficulty levels and illustrate its setting (see Figure S1).

## Game 1

In the first game, the child had to run in slalom around six pylons (i.e., three red and three blue pylons) holding a ball (ø 16cm) in their hands. On the way back, the child had to dribble the ball straight ahead alongside the pylons with their feet. After completing three correct rounds (i.e., not missing a pylon, not losing the ball while dribbling), the child proceeded to the second difficulty level and was instructed to touch the floor with the ball in front of the blue pylons, and throw the ball in the air and catch it again in front of the red pylons. On the way back, the child had to dribble the ball straight ahead in between the pylons.

## Game 2

In this game, the child had to run through a coordination ladder, and on the way back, balance on a rope (3m long, 1cm wide) arranged in slalom. After completing three correct rounds (i.e., not stepping on the coordination ladder, not stepping outside the rope), the child proceeded to the second difficulty level and was instructed to jump through the coordination ladder. Specifically, the child had to jump with one foot into three ladder fields marked with a red slip of paper (19cm x 11cm) each and jump with two feet into the non-marked ladder fields. On the way back, the child should again balance on the rope.

## Game 3

In the third game, the child had to bounce a ball (ø 16cm) with two hands into three different red-marked fields of the coordination ladder while running alongside it. At the end of the ladder, the child had to turn around and throw the ball into a hula hoop (Ø 52cm) lying on the floor 2m away. If the child completed three correct rounds (i.e., not losing the ball after bouncing it, not throwing it outside the hula hoop), the second difficulty level was introduced. Here, the child had to again bounce the ball into the red marked ladder fields, but on the way back, they had to throw the ball into a hula hoop 3m away.

## Game 4

In this game, the child was instructed to run from one red slip of paper (19cm x 11cm) to another red slip of paper and bend down and touch the slips of paper with his or her hands. The three slips of paper were posited in a line on the floor and were each 1m apart. They were surrounded by two jumping ropes (3m long each) forming a pathway. On the way back, the child should jump sideways next to this pathway. After completing three correct rounds (i.e., touching all slips of paper, not jumping forwards on the way back), the child entered the second difficulty level and was instructed to perform a high straight jump. The experimenter first practiced two jumps with the child and emphasized that the child had to bend his knees and hand his arms down and then jump straight upwards while lifting both arms and hands upwards. Then the child should run from one slip of paper to the other and perform a high straight jump at each slip of paper instead of just touching it. On the way back, the child had to jump sideways again.

| 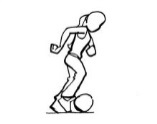 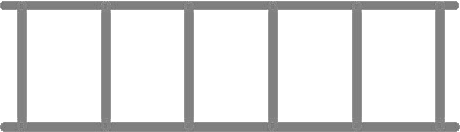 |
| --- |


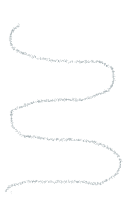

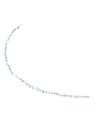

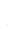

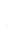

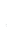


Game 2

Game 1

Game 3


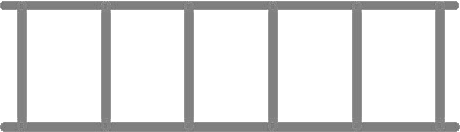


2-3m


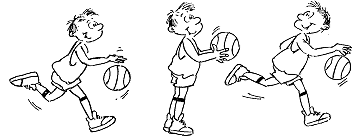


Game 4

**Figure S1**. Settings for the different games in the motor coordination intervention.

**Table S2. Criteria for passing Level 1 and Level 2 of each motor coordination game**

|  | Level 1 | Level 2 |
| --- | --- | --- |
| Game 1 | - Slalom running around each pylon - *Return*: Dribble the ball straight ahead alongside the pylons | - Touch the floor with the ball at each blue pylon - Throw the ball in the air and catch it again at each red pylon   *Return*: Dribble the ball straight ahead in between the pylons |
| Game 2 | - Step into each field of the coordination ladder without touching it - *Return*: Balance correctly on the rope | - Jump with both feet in non-marked ladder fields - Jump with one foot in each field of the coordination ladder marked with a red card - *Return*: Balance correctly on the rope |
| Game 3 | - Bounce and catch the ball in each field marked with a red card (without losing the ball) - *Return*: Throw the ball from the end of the coordination ladder and hit the tire in 2 m distance | - Bounce and catch the ball in each field marked with a red card (without losing the ball) - *Return*: Throw the ball from the end of the coordination ladder and hit the tire in 3 m distance |
| Game 4 | - Run between ropes and touch each red card with your hand - *Return*: Correct execution of sideways hopping | - Run between the ropes and correctly execute the high straight jump at each point marked with a red card - *Return*: Correct execution of sideways hopping |
