# Supplementary material for: Acute effects of a motor coordination intervention on executive functions in kindergartners: a proof-of-concept randomized controlled trial
Source: Pilot Feasibility Stud. 2022 Aug 17;8:185. doi: 10.1186/s40814-022-01125-w (PMC9382724; doi:10.1186/s40814-022-01125-w)
Supplement: Supplementary file 3 — Additional file 3. Affect measure. The assessment of affect is described in detail. [file 40814_2022_1125_MOESM3_ESM.docx]

# Additional file 3 - Affect

Affect measure

To assess the children’s current affect before and after the intervention/ control condition, we used the Self-Assessment Manikin Scale (original [1], German version [2]) with two items representing (a) valence (unhappy, bad-humored to happy, good-humored) and (b) arousal (calm, relaxed to excited, awake) by age-appropriate adjectives [3]. Children were instructed to indicate their current affect by pointing to one of the 5 manikins showing varying expressions that best matched their affect (5-point bipolar scale). The manikins in the first row are labeled as happy and good-humored on the left side or unhappy and bad-humored on the right side. The manikins in the second row are labeled as excited and awake on the left side and calm and relaxed on the right side. The levels in between are directly labeled by highlighting the degree of these affect states.


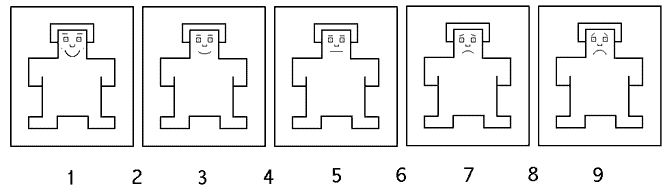


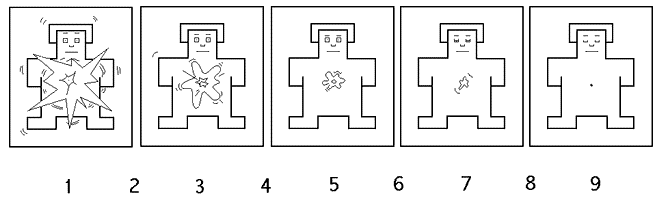


References

1. Bradley MM, Lang PJ. Measuring emotion: The self-assessment manikin and the semantic differential. J Behav Ther Exp Psychiatry. 1994;25:49–59. doi:10.1016/0005-7916(94)90063-9.

2. Conzelmann A, McGregor V, Pauli P. Emotion regulation of the affect-modulated startle reflex during different picture categories. Psychophysiology. 2015;52:1257–62. doi:10.1111/psyp.12450.

3. Sharp C, van Goozen S, Goodyer I. Children's subjective emotional reactivity to affective pictures: gender differences and their antisocial correlates in an unselected sample of 7-11-year-olds. J Child Psychol Psychiatry. 2006;47:143–50. doi:10.1111/j.1469-7610.2005.01464.x.
