# Supplementary material for: Acute effects of a motor coordination intervention on executive functions in kindergartners: a proof-of-concept randomized controlled trial
Source: Pilot Feasibility Stud. 2022 Aug 17;8:185. doi: 10.1186/s40814-022-01125-w (PMC9382724; doi:10.1186/s40814-022-01125-w)
Supplement: Supplementary file 4 — Additional file 4. Motor coordination experience. Questionnaire items to assess motor coordination experience are described in detail. [file 40814_2022_1125_MOESM4_ESM.docx]

Additional file 4 - Motor coordination experience

To assess motor coordination experience, we used the Questionnaire of Physical Activity in Preschool Children [1],which consisted of the following questions:

1. Do you or other persons take your child for swimming?
2. How often does your child climb on trees, wall bars or similar?
3. How often does your child play with a ball?
4. How often does your child play tag?
5. How often does your child ride a bike or use a kick scooter?
6. How often does your child skate (roller skates, inline skates)?
7. Does your child attend a sports club or sports group?

Bayer and colleagues categorized the sum score into three equidistant categories of low, moderate and high levels of physical activity. However, we used the sum score itself to avoid reducing the available information by further categorization and since the sum score followed a normal distribution in our sample. Moreover, we reran all analyses with the categorized score and the results did not change.

References

1. Bayer O, Jarczok M, Fischer J, Kries R von, Bock F de. Validation and extension of a simple questionnaire to assess physical activity in pre-school children. PHN. 2012;15:1611–9. doi:10.1017/S1368980012001243.
