# Supplementary material for: Acute effects of a motor coordination intervention on executive functions in kindergartners: a proof-of-concept randomized controlled trial
Source: Pilot Feasibility Stud. 2022 Aug 17;8:185. doi: 10.1186/s40814-022-01125-w (PMC9382724; doi:10.1186/s40814-022-01125-w)
Supplement: Supplementary file 5 — Additional file 5. Further analyses on the acute effectiveness of the conditions. Regression analyses of the conditions on executive function performances are described in detail. [file 40814_2022_1125_MOESM5_ESM.docx]

**Additional file 5 - Further analyses on the acute effectiveness of the conditions**

***Objective***

This pilot study further analysed whether there are differential intervention effects depending on the outcome measure (near-transfer vs. far-transfer measure of EF) and whether children’s previous motor coordination experiences moderate the intervention effects.

***Methods***

Preliminary intervention effects were analyzed on each outcome variable (i.e., HTKS, DNS) separately by running linear regression models with the condition as predictor and controlling for background variables (age, sex, motor coordination experience and self-control). This further analyses addressed differences in intervention effects depending on the outcome measure (i.e. near-transfer measure of EF = HTKS vs. far-transfer measure of EF = DNS) by comparing standardized regression coefficients. In addition, we included levels of motor coordination experience as predictor and moderator of the intervention effects. All predictors were centered at the grand mean or dummy-coded, we screened for multicollinearity between predictors. The Johnson-Neyman technique was used to probe for significant interactions. The level of significance was set to *p* < .05 for all analyses.

***Results***

*Head-toes-knees-shoulders task*

There was a significant main effect of age indicating higher performance for older children (β = 0.68; *∆R²* = .25; see Table A5). Neither sex nor parent-reported self-control predicted children’s HTKS performance. There was no multicollinearity between predictors. Exploratory analyses of level of motor coordination experience showed a significant interaction effect with condition (β = 0.25). Experimental condition was significantly related to HTKS performance when the level of motor coordination experience was 2.12 *SD* below the mean or 1.60 *SD* above the mean level of motor coordination experience. Experimental condition was not significantly related to HTKS performance when the children’s level of motor coordination experience was at the mean (*p* >.05). This interaction effect (β = 0.25; *∆R²* = .03) was the second most influential predictor (after age, β = 0.68) of HTKS performance.

*Day-night Stroop-like task*

There was a main effect of age indicating that children exhibited improved DNS performance with increasing age (β = 0.37; ∆*R²* = .25). In addition, we found a main effect of self-control such that children with higher parent-reported self-control exhibited better DNS performance than children with lower self-control (β = 0.20; ∆*R²* = .04). Sex did not predict children’s performance. There was no multicollinearity between predictors. Exploratory analyses of children’s level of motor coordination experience showed no significant moderating effect.

***Discussion***

Exploratory analyses revealed differential intervention effects depending on children’s motor coordination experiences and the intervention condition on a near transfer measure, but not on a far transfer measure of EFs. Children with very low levels of motor coordination experience performed worse in one of two EF tasks after participating in the motor coordination intervention compared to the control condition, whereas children with very high levels of motor coordination experience performed better in this task after the motor coordination intervention compared to the control condition.

**References**

Johnson, P. O., & Fay, L. C. (1950). The Johnson-Neyman technique, its theory and application. *Psychometrika*, *15*(4), 349–367. https://doi.org/10.1007/BF02288864

| Table A5.  *Regression analyses: Predicting executive functions performance* | | | | | | | | | | | | | | | | | | | | | | | | |
| --- | --- | --- | --- | --- | --- | --- | --- | --- | --- | --- | --- | --- | --- | --- | --- | --- | --- | --- | --- | --- | --- | --- | --- | --- |
|  | | | | *n* | *B* | *SE(B)* | | | 95% CI | | | | β | | *t* | | *p* | *F* | *df* | *p* | *R²* | *adj.R²* | *∆R²* |  |
| *Head-toes-knees-shoulders task* | | | |  |  |  | | |  | |  | |  | |  | |  |  |  |  |  |  |  |  |
| Overall model | | | | 94 |  |  | | |  | |  | |  | |  | |  | 17.48 | 6,87 | **<.001** | .55 | .52 |  |  |
| Age | | | |  | 0.65 | 0.07 | | | 0.51 | | 0.90 | | 0.68 | | 9.15 | | **<.001** |  |  |  |  |  | .22 |  |
| Sex | | | |  | 2.77 | 1.88 | | | -0.96 | | 6.50 | | 0.11 | | 1.47 | | .144 |  |  |  |  |  |  |  |
| Self-control | | | |  | 0.16 | 0.11 | | | -0.05 | | 0.37 | | 0.12 | | 1.55 | | .124 |  |  |  |  |  |  |  |
| Condition | | | |  | 0.13 | 1.80 | | | -3.44 | | 3.71 | | 0.01 | | 0.07 | | .941 |  |  |  |  |  |  |  |
| Motor coordination experience | | | |  | -0.77 | 0.42 | | | -1.60 | | 0.07 | | -0.20 | | -1.83 | | .071 |  |  |  |  |  |  |  |
| Condition*Motor coordination experience | | | |  | 1.28 | 0.57 | | | 0.14 | | 2.42 | | 0.25 | | 2.23 | | **.028** |  |  |  |  |  | .03 |  |
|  |  |  |  | | | | | | | | | | | | | | | | | | | | | |
| *Day-night Stroop-like task* | | | |  |  | |  |  | |  | |  | |  | |  | |  |  |  |  |  |  |  |
| Overall model | | | | 97 |  | |  |  | |  | |  | |  | |  | | 8.44 | 6,90 | **<.001** | .36 | .32 |  |  |
| Age | | | |  | 0.75 | | 0.13 | 0.50 | | 1.00 | | 0.52 | | 5.95 | | **<.001** | |  |  |  |  |  | .25 |  |
| Sex | | | |  | 3.17 | | 3.03 | -3.94 | | 9.73 | | 0.09 | | 0.96 | | .340 | |  |  |  |  |  |  |  |
| Self-control | | | |  | 0.43 | | 0.19 | 0.05 | | 0.80 | | 0.20 | | 2.24 | | **.028** | |  |  |  |  |  | .04 |  |
| Condition | | | |  | -0.62 | | 3.16 | -6.90 | | 5.65 | | -0.02 | | -0.20 | | .844 | |  |  |  |  |  |  |  |
| Motor coordination experience | | | |  | 0.02 | | 0.72 | -1.42 | | 1.45 | | 0.01 | | 0.02 | | .981 | |  |  |  |  |  |  |  |
| Condition*Motor coordination experience | | | |  | 0.07 | | 1.01 | -1.95 | | 2.08 | | 0.01 | | 0.07 | | .949 | |  |  |  |  |  |  |  |
| *Note*. Age, self-control and motor coordination experience are centered at the grand mean; dummy-coded variables: Sex with boys: 0, girls: 1, condition with control: 0, intervention: 1, in bold if *p* < 0.05. | | | | | | | | | | | | | | | | | | | | | | | | |
